# ECG Sensor Design Assessment with Variational Autoencoder-Based Digital Watermarking

**DOI:** 10.3390/s25072321

**Published:** 2025-04-05

**Authors:** Chih-Yu Hsu, Chih-Yin Chang, Yin-Chi Chen, Jasper Wu, Shuo-Tsung Chen

**Affiliations:** 1 School of Transportation, Fujian University of Technology, Fuzhou 350118, China; 61201903@fjut.edu.cn; 2 Department of Medical Informatics, Chung Shan Medical University, Taichung 40201, Taiwan; chloe19991023@gmail.com (C.-Y.C.); chenkili0216@gmail.com (Y.-C.C.); 3 Kang Chiao International School, Linkou Campus, New Taipei City 244, Taiwan; ls13056@stu.kcislk.ntpc.edu.tw; 4 Department of Electronic Engineering, National Kaohsiung University of Science and Technology, Kaohsiung City 807618, Taiwan

**Keywords:** variational AutoEncoder, Fourier-simulated ECG dataset, latent variable space, watermarking technology

## Abstract

Designing an ECG sensor circuit requires a comprehensive approach to detect, amplify, filter, and condition the weak electrical signals produced by the heart. To evaluate sensor performance under realistic conditions, diverse ECG signals with embedded watermarks are generated, enabling an assessment of how effectively the sensor and its signal-conditioning circuitry handle these modified signals. A Variational Autoencoder (VAE) framework is employed to generate the watermarked ECG signals, addressing critical concerns in the digital era, such as data security, authenticity, and copyright protection. Three watermarking strategies are examined in this study: embedding watermarks in the mean (μ) of the VAE’s latent space, embedding them through the latent variable (z), and using post-reconstruction watermarking in the frequency domain. Experimental results demonstrate that watermarking applied through the mean (μ) and in the frequency domain achieves a low Mean Squared Error (MSE) while maintaining stable signal fidelity across varying watermark strengths (α), latent space dimensions, and noise levels. These findings indicate that the mean (μ) and frequency domain methods offer robust performance and are minimally affected by changes in these parameters, making them particularly suitable for preserving ECG signal quality. By contrasting these methods, this study provides insights into selecting the most appropriate watermarking technique for ECG sensor applications. Incorporating watermarking into sensor design not only strengthens data security and authenticity but also supports reliable signal acquisition in modern healthcare environments. Overall, the results underscore the effectiveness of combining VAEs with watermarking strategies to produce high-fidelity, resilient ECG signals for both sensor performance evaluation and the protection of digital content.

## 1. Introduction

**Figure 1:**
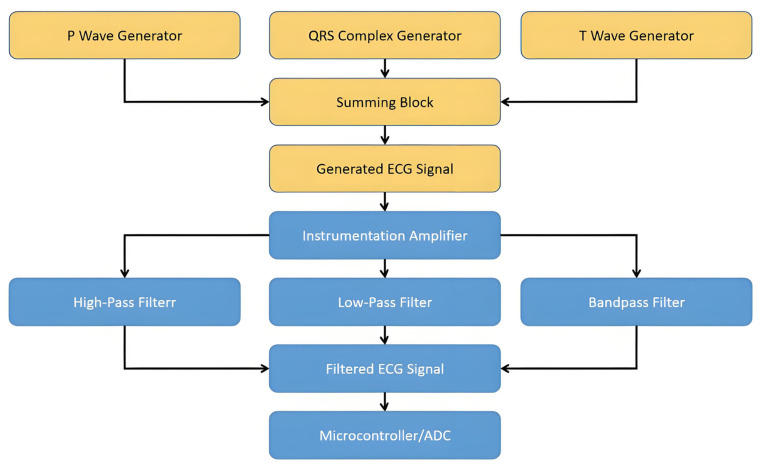
Functional Block Diagram of ECG signal processing.

Designing an ECG sensor circuit involves creating a circuit that can detect, amplify, filter, and condition the weak electrical signals generated by the heart. Here, basic functional blocks include an ECG sensor circuit. The Functional Block Diagram for the ECG sensor is shown in Figure 1. Designing an ECG sensor circuit necessitates a comprehensive approach that involves detecting, amplifying, filtering, and conditioning the weak electrical signals produced by the heart. This area of research has garnered significant attention in recent years, with various techniques being proposed to evaluate sensor performance under realistic conditions. Several domestic and international groups, including Guangli Li’s team [1,2,3], have contributed significantly to this field by developing significant performance gains on edge devices, and extensive validation across various DNN architectures and datasets confirms that these integrated techniques enable efficient, scalable, and practical deep learning applications. In this paper, we build upon these existing works by employing a Variational Autoencoder (VAE) framework for generating watermarked ECG signals, addressing critical concerns in data security, authenticity, and copyright protection.

The electrodes are connected to the instrumentation amplifier, followed by the high-pass, low-pass, and band-pass filters. The circuit conditions the ECG signal before feeding it into a microcontroller or ADC. To evaluate the performance of sensors in ECG signal processing, the generation of various ECG signals is essential. This can help test how well the sensors and signal conditioning circuit handle different types of ECG data.

The performance of sensors is significantly influenced by the specifications of the filters they use. For example, we can consider a sensor system that incorporates the following filters:

- High-Pass Filter: a cutoff frequency of 1.0 Hz
- Low-Pass Filter: a cutoff frequency of 10.0 Hz
- Band-Pass Filter: a low cutoff frequency of 5.0 Hz and a high cutoff frequency of 20.0 Hz

These filter specifications define how the sensor processes and conditions the signal, affecting its overall performance.

**Figure 2:**
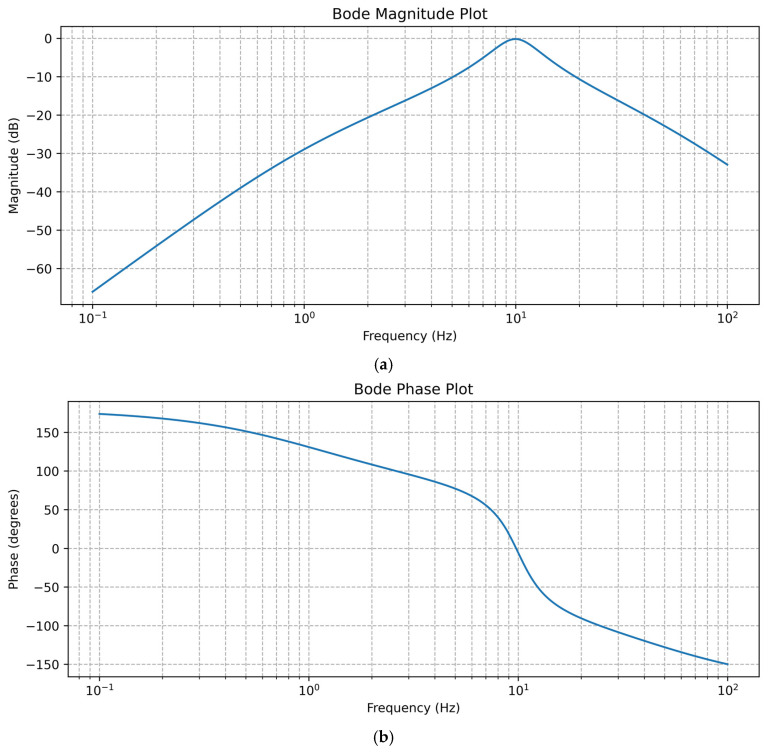
The plots represent (**a**) the Bode magnitude and (**b**) the phase response of a system consisting of high-pass, band-pass, and low-pass filters.

Figure 2 shows decibels (dB) plotted on the vertical axis, with frequency (Hz) on the horizontal axis (on a logarithmic scale). The magnitude plot indicates a small range of attenuation (~2 dB change) over three orders of magnitude in frequency, while the **phase plot** shows a transition of a few hundred degrees (with unwrapping). This is characteristic of a **very low-gain (high-attenuation) system** or possibly part of a **precision analog filtering stage** intended to suppress signals well below the −100 dB mark across the given frequency band.

Electrocardiogram (ECG) signals capture the cardiac electrical activity and play a pivotal role in clinical diagnosis, patient monitoring, and healthcare research. With the escalating adoption of digital health records and the emergence of telemedicine, ensuring the security and integrity of ECG data has become a paramount concern. While watermarking techniques have been extensively studied for images and audio, their application to ECG signals is relatively recent. Initial endeavors have concentrated on embedding or identifying patient information into the temporal or spectral domain of the ECG waveform, with the objective of preserving clinical utility while safeguarding data. Expanding upon these foundational methods, adaptive watermarking strategies have begun to surface, adapting embedding parameters based on ECG characteristics to heighten both resilience and imperceptibility [4,5,6,7,8,9,10,11,12,13,14,15,16].

As digital health infrastructure continues to evolve, researchers have explored advanced signal processing and machine learning techniques to strengthen watermarking strategies. Deep Neural Networks (DNNs) [15], Convolutional Neural Networks (CNNs) [17,18,19,20,21,22], Generative Adversarial Networks (GANs), and Variational Autoencoders (VAEs) have introduced new ways to blend watermarks seamlessly with ECG data. These generative models learn intricate signal patterns, enabling the insertion of watermarks that not only remain imperceptible to human observation but also endure common signal degradation. In particular, GANs and VAEs have proven effective at creating watermarks that closely resemble original ECG signals, reducing perceptual distortion and reinforcing data security.

Although these advancements have led to the emergence of more durable and inconspicuous watermarks, challenges still endure. Striking a delicate balance between watermark robustness and signal fidelity remains paramount, as excessive embedding can compromise the diagnostic precision of ECG data. Furthermore, attaining interoperability and standardization is imperative for the practical integration of these techniques into clinical settings. With the continuous progression of artificial intelligence, the incorporation of these innovations into watermarking procedures shows potential for further enhancing resilience, efficacy, and the ethical management of confidential patient data.

Given these evolving requirements, this work investigates a watermarking framework that combines digital watermarking techniques with Variational Autoencoders (VAEs) to produce and protect ECG data. VAEs serve as powerful generative tools capable of synthesizing realistic ECG signals, which can then incorporate watermark information in different ways. For instance, the watermark can be embedded directly into the latent space—either through the mean parameter (μ) or the latent vector (z)—or inserted post-reconstruction in the frequency domain. Each approach offers unique advantages and trade-offs: embedding watermarks in the latent space can provide strong invisibility but may impact signal quality, whereas post-reconstruction watermarking can preserve signal fidelity yet require more complex extraction processes.

By meticulously scrutinizing and contrasting these embedding methodologies, this research tackles the increasing demand to safeguard ECG data against tampering and unauthorized dissemination. The outcomes underscore the adaptability of VAEs and watermarking techniques to various application scenarios, effectively balancing imperceptibility, robustness, and computational intricacies. This introduction sets the stage for the ensuing sections, which intricately outline the methodological framework, experimental configurations, and pivotal discoveries that underscore the advantages and obstacles in harnessing VAEs for ECG watermarking. Ultimately, this study endeavors to steer forthcoming inquiries toward scalable, secure, and efficient watermarking solutions for medical data administration in contemporary healthcare settings.

## 2. Variational Autoencoders (VAEs)

**Figure 3:**
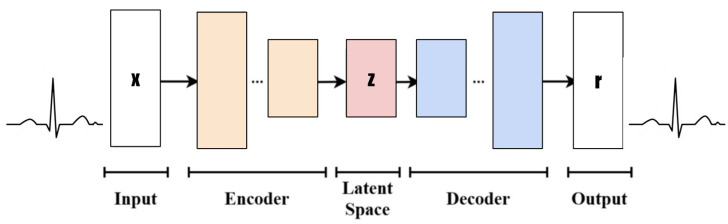
The architecture of the VAE.

Variational autoencoders (VAEs) are a generative model that combines autoencoders and variational inference. They are part of the families of probabilistic graphical models and variational Bayesian methods [23]. In machine learning, a Variational Autoencoder (VAE) is an artificial neural network architecture introduced by Diederik P. Kingma and Max Welling [24]. Its mathematical foundation mainly includes probabilistic graphical models, variational inference, and autoencoders. The following diagram, as shown in Figure 3, is an introduction to VAE’s architecture.

The diagrams in Figure 3 are described and explained as follows.

- Input: Design a rectangle or ellipse with “Input” inside. The data type is the time-series ECG signal (x) and the dimension (1, 100).
- Encoder: Use multiple rectangles or trapezoid stacks to represent the encoder layer. Each rectangle can be simply marked with “Enc Layer” or a specific layer name (such as the convolution layer, pooling layer, etc.), and arrows indicate the data flow direction.
- Latent Space: This is represented by a circle or oval, with “Latent Space” marked inside. You can add some small dots or cloud patterns to symbolize potential representation.
- Decoder: This is similar to the encoder design but in the opposite direction, and it means decoding data from the latent space back to the original or target domain.
- Output: The type of output data (reconstructed x) and dimension (1, 100) are labeled “Output”.

### 2.1. VAE Mathematical Description

A Variational Autoencoder (VAE) consists of encoder and decoder networks, with a latent representation in between.

**Encoder**

The encoder maps the input ECG segment x to two outputs: the mean vector μ and the log variance vector $log(\sigma^{2})$. Typical architectures include convolutional layers (for 1D signals) or fully connected layers.

**Latent Space**

Latent space uses the reparameterization trick to sample a latent variable z from the distribution $N\left(\mu ,\sigma^{2}\right)$:$\;z=\mu +\epsilon \cdot \sigma ,\epsilon ∼N\left(0,I\right).$

**Decoder**

The decoder maps the latent variable z back to a reconstructed ECG signal $\hat{x}$.

Reconstruction quality is measured using a loss function (e.g., Mean Squared Error, MSE).

**Training Objective**

**Reconstruction Loss** (e.g., MSE or MAE) is between x and $\hat{x}$.

**KL Divergence** is used to regularize the learned distribution relative to a standard Gaussian, $L_{\mathrm{VAE}}=L_{\mathrm{Recon}}+\beta \cdot L_{\mathrm{KL}},$ where β balances reconstruction fidelity and latent space regularization.

**Probabilistic graphical model**

VAE is a generative model based on the probabilistic graphical model. Suppose we have an observed variable $\left(x\right)$ and a latent variable $\left(z\right)$, then we can define the joint probability distribution as follows:

(1) $$p\left(x,z\right)=p\left(x|z\right)p\left(z\right)$$

where $p\left(z\right)\;$is the prior distribution of the latent variable, which is usually assumed to be the standard normal distribution $N\left(0,1\right).$ $p\left(x|z\right)$ is the conditional probability distribution of the observed variable given the hidden variable.

### 2.2. Variational Reasoning

In VAE, we wish to maximize the log-likelihood of the observed data, $logp\left(x\right):$

(2) $$logp\left(x\right)=log\int p\left(x,z\right)\;dz=log\int p\left(x|z\right)p\left(z\right)\;dz$$

Due to the difficulty in directly computing this integral, we employed variational inference to approximate it. An approximate posterior distribution *q*(*z*|*x*) was introduced, and Jensen’s inequality was utilized to derive the evidence lower bound (ELBO):

The ELBO provides a tractable objective function that, when optimized, leads to an approximation of the true posterior distribution that is both tractable and useful for inference and generation tasks. By maximizing the ELBO, we can essentially minimize the difference between the true posterior *p*(*z*|*x*) and the approximate posterior $q\left(z|x\right)$, while also optimizing good reconstructions of the input data *x* and using Jensen’s inequality to obtain the evidence lower bound (ELBO):

(3) $$logp\left(x\right)\geq E_{q\left(z|x\right)}\left[logp\left(x|z\right)\right]-KL\left(q\left(z|x\right)∥p\left(z\right)\right)$$

where $KL\left(q\left(z|x\right)∥p\left(z\right)\right)$ is between $q\left(z|x\right)$ and $p\left(z\right)$ *KL* Divergence. We can maximize this lower bound, which is represented by the following:

(4) $$L\left(x\right)=E_{q\left(z|x\right)}\left[logp\left(x|z\right)\right]-KL\left(q\left(z|x\right)∥p\left(z\right)\right)$$

### 2.3. Autoencoder

VAE uses two neural networks to parameterize $p\left(x|z\right)$ and $q\left(z|x\right).\;\;The\;q\left(z|x\right)$ **encoder (inference network) uses the parameters** $\left(\mu \left(x\right)\right)$ **and** $\left(\sigma {\left(x\right)}^{2}\right)$ **that map the input data *x* to the latent variable *z*, i.e.,**

(5) $$q\left(z|x\right)=N\left(z;\mu \left(x\right),\sigma {\left(x\right)}^{2}\right)$$

**The decoder (generator network) maps the latent variable *z* back to the data space and generates** $p\left(x|z\right).$

The specific steps of the autoencoder are as follows: The first encode passes the input x through the encoder network map to the mean and variance of the latent variable. Then, the re-parameterization technique is used to calculate the gradient. The re-parameterization technique is used to calculate the gradient from $q\left(z|x\right)$ medium sampling. The sample from the standard normal distribution, ϵ, is then computed $z=\mu \left(x\right)+\sigma \left(x\right)⊙\epsilon$. Then, for the decode, *z* is mapped back to data space through the decoder network to obtain the reconstructed x. 

### 2.4. Loss Function

The loss function of a Variational Autoencoder (VAE) comprises two main parts:

- Reconstruction Error: This measures the difference between the reconstructed x and the original x. Commonly used metrics for this purpose are the Mean Squared Error (MSE) or Binary Cross-Entropy (BCE), depending on the nature of the data being reconstructed.
- KL Divergence: This measures the difference between the approximate posterior distribution $q\left(z|x\right)$ and the prior distribution $p\left(z\right)$. KL Divergence, also known as Kullback–Leibler Divergence, is a measure of how one probability distribution diverges from another. In the context of VAEs, minimizing this divergence encourages the approximate posterior to resemble the prior distribution, typically a simple distribution like a multivariate Gaussian, facilitating the generation of new samples.

The total loss function is as follows:

(6) $$L=E_{q\left(z|x\right)}\left[logp\left(x|z\right)\right]-KL\left(q\left(z|x\right)∥p\left(z\right)\right)$$

## 3. Methodology

This section describes the experimental setup, parameters, and procedures for embedding watermarks in ECG signals using Variational Autoencoders (VAEs). It consolidates the three primary watermarking methods—embedding watermarks into the latent mean (*μ*), embedding them via the latent variable (*z*), and post-reconstruction embedding in the frequency domain—while providing step-by-step explanations of both embedding and extraction processes.

### 3.1. Data Preparation and Preprocessing

A suitable ECG dataset was sourced, ensuring that the data included all relevant features used for diagnosis. The sampling rate ranged between 200 and 500 Hz. If necessary, long ECG recordings were split into segments of fixed length (e.g., 2–5 s) to create uniform inputs for the VAE. ECG segments were normalized or scaled (e.g., to [−1, 1] or a mean of 0 and standard deviation of 1). This step helps stabilize VAE training and ensures watermark signals are added consistently across samples. The dataset was then split into training (∼80%), validation (∼10%), and testing (∼10%) sets. The validation set helps tune hyperparameters, while the test set provides unbiased performance metrics for the final model.

#### Fourier-Simulated ECG

**Figure 4:**
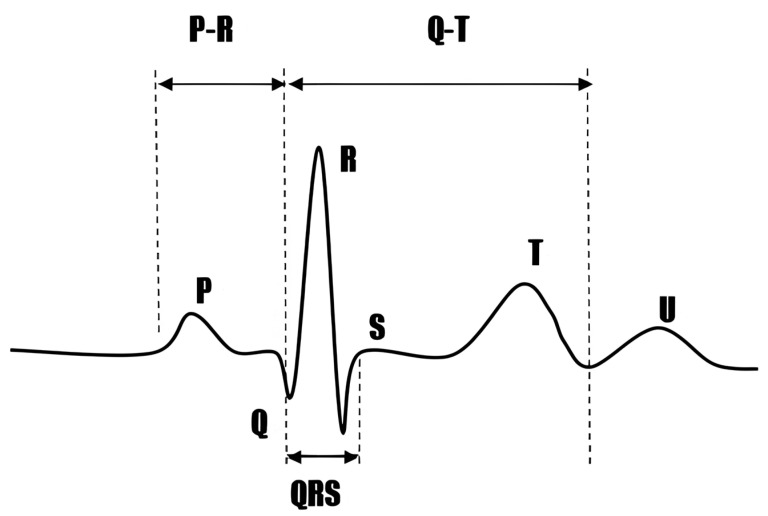
The simulated electrocardiogram (ECG) [27,28,29,30].

The Simulated Electrocardiogram (ECG) [25,26,27,28,29,30], as shown in Figure 4, is a graphical representation of the electrical activity of the heart, which plays a vital role in medicine.

The ECG consists of several distinct components interconnected by the isoelectric line. The isoelectric line is important, and actual ECG recordings often show some baseline shift, even after filtering. The isoelectric line appears as a relatively flat baseline on the ECG, signifying periods when the heart exhibits no or minimal electrical activity. Below are the primary components of the ECG and their relationship to the isoelectric line:

- P Wave: The P wave represents atrial depolarization, with the electrical signal preceding atrial muscle contraction. It typically lies above the isoelectric line, and its morphology and amplitude can provide insights into atrial size and conduction abnormalities.
- PR Interval: The PR interval measures the time from the onset of the P wave to the start of the QRS complex, reflecting the duration of electrical signal conduction from the atria to the ventricles. During the PR interval, the ECG typically displays the isoelectric line, indicating that ventricular depolarization has not yet begun.
- QRS Complex: The QRS complex represents ventricular depolarization, which is the most prominent and substantial part of the ECG. It consists of three waves (Q, R, and S), though not every QRS complex fully exhibits all three. The isoelectric line preceding the QRS complex signifies the absence of ventricular depolarization, while the subsequent isoelectric line may indicate the completion or imminent onset of ventricular repolarization.
- ST Segment: The ST segment connects the end of the QRS complex to the beginning of the T wave, reflecting the early phase of rapid ventricular repolarization. In normal conditions, the ST segment should be level with or slightly below the isoelectric line without significant deviation. Changes in the ST segment (e.g., elevation or depression) are crucial indicators of myocardial ischemia or infarction.
- T Wave: The T wave represents the slow repolarization of the ventricles and the electrical signal as ventricular muscle cells gradually return to their resting potential after contraction. The direction and amplitude of the T wave can be influenced by various factors, including myocardial metabolic status, medication effects, and electrolyte balance.
- QT Interval: The QT interval measures the time from the onset of the QRS complex to the end of the T wave, encompassing the total duration of ventricular depolarization and complete repolarization. The prolongation or shortening of the QT interval may be associated with arrhythmia, electrolyte disturbances, or medication effects.
- U Wave: Following the T wave, with an interval of 0.02 to 0.04 s, the U wave is wide, low, and typically has an amplitude below 0.05 millivolts and a duration of approximately 0.20 s.

On the ECG, the isoelectric line not only connects the various waveform components but also serves as a crucial reference for assessing waveform normality and identifying abnormal changes. By observing the stability of the isoelectric line and the positional changes in waveforms relative to it, physicians can preliminarily determine whether the heart’s electrical activity is normal or indicative of potential cardiac diseases.

### 3.2. Watermarking Modes in VAEs

Watermarking involves embedding imperceptible yet detectable information into ECG signals to verify data integrity or ownership. This can be accomplished in pre-reconstruction or post-reconstruction phases, each with distinct trade-offs.

#### 3.2.1. Pre-Reconstruction: Embedding into the Mean (μ)

**Embedding Process**

(a) **Encoding**: Pass the ECG segment x through the encoder to obtain μ and $log(\sigma^{2})$.
(b) **Watermark Integration**: Concatenate or add a scaled watermark w to the mean$,\;\mu_{w}=\mu +\alpha \cdot w,$ where α is the embedding strength.
(c) **Reparameterization**: Sample the latent variable z using $\mu_{w}$ and σ:$z=\mu_{w}+\epsilon \cdot \sigma ,\epsilon ∼N\left(0,I\right).$
(d) **Decoding**: Pass z to the decoder to generate the watermarked ECG signal $\hat{x}$.

**Extraction Process**

(a) **Decoding**: When verifying, encode the suspected watermarked ECG $\hat{x}$ again to obtain $\mu_{w}$.
(b) **Watermark Retrieval**: Compute the difference between the original μ (if known or stored) and $\mu_{w}$:$w=\frac{\mu_{w}-\mu }{\alpha }.$ This reverses the embedding operation and recovers the watermark.

**Notes and Trade-offs**

(a) **Advantages**: Advantages include strong invisibility and minimal additional computation.
(b) **Disadvantages**: Disadvantages include the fact that it requires knowledge of the original mean μ for exact extraction; a large α could degrade reconstruction quality.

#### 3.2.2. Pre-Reconstruction: Embedding Through the Latent Variable (z)

**Embedding Process**

(a) Encoding: Obtain μ and $log(\sigma^{2})$ from the encoder.
(b) Reparameterization: Sample the initial latent variable $z_{\mathrm{original}}=\mu +\epsilon \cdot \sigma$.
(c) Watermark Integration: Add a scaled watermark w to $z_{\mathrm{original}}$:$z_{w}=z_{\mathrm{original}}+\alpha \cdot w.$
(d) Decoding: Pass $z_{w}$ to the decoder to generate the watermarked ECG signal $\hat{x}$.

**Extraction Process**

(a) Re-encode: Encode the watermarked ECG to obtain $z_{w}$.
(b) Retrieval: Subtract the original $z_{\mathrm{original}}$ from $z_{w}$ (if $z_{\mathrm{original}}$ is available) or compare it with known references: $w=\frac{z_{w}-z_{\mathrm{original}}}{\alpha }.$

**Notes and Trade-offs**

(a) Advantages: Advantages include the fact that it preserves μ without modification, potentially simplifying certain aspects of the retrieval.
(b) Disadvantages: Disadvantages include that the process must handle storing or recomputing the original $z_{\mathrm{original}}$; increasing α may introduce more distortion.

#### 3.2.3. Post-Reconstruction: Frequency-Domain Watermarking

**Embedding Process**

(a) **VAE Reconstruction**: Generate the reconstructed ECG $\hat{x}$ from a latent vector.
(b) **Fourier Transform**: Convert $\hat{x}$ to the frequency domain (e.g., via Fast Fourier Transform, FFT).
(c) **Watermark Insertion**: Modify selected frequency coefficients based on the watermark bits. For instance,
  ▪ If watermark bit = 1, increase the magnitude by α.
  ▪ If watermark bit = 0, decrease the magnitude by α.

(d) **Inverse Transform**: Apply the inverse FFT to obtain a watermarked ECG signal in the time domain.

**Extraction Process**

(a) **Fourier Transform of Watermarked ECG**: Convert the watermarked signal back to the frequency domain.
(b) **Compare Coefficients**: Check how each targeted coefficient changes relative to a reference (either an original or a threshold-based approach).
(c) **Reconstruct Watermark Bits**: If a coefficient is higher than expected, interpret that as bit 1; otherwise, interpret it as bit 0.

**Notes and Trade-offs**

(a) **Advantages**: Advantages include the fact that there is generally minimal impact on time-domain clinical features if embedding is performed carefully; there is robust to minor time-domain noise.
(b) **Disadvantages**: Disadvantages include more computational steps (FFT and IFFT); detection requires frequency-domain analysis and may be vulnerable to frequency-specific attacks (e.g., notch filtering).

## 4. Experimental Results and Discussions

### 4.1. Simulated Dataset

**Figure 5:**
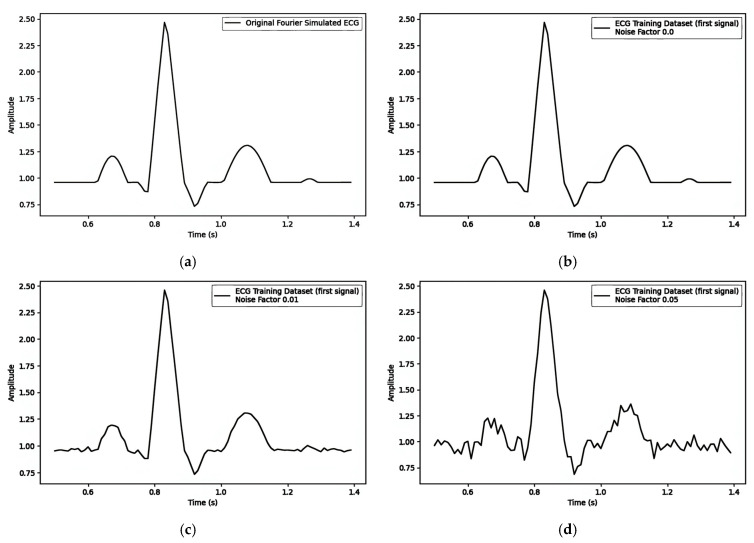
The simulated ECG (**a**) original signal with the noise factors (**b**) 0.00, (**c**) 0.01, and (**d**) 0.05.

A training dataset built with Fourier ECG signals, as shown in Figure 5a, with varying noise factors was generated. These datasets were then used to train VAE (Variational AutoEncoder) models. Each trained VAE model was utilized to generate ECG signals, as demonstrated in Figure 5 below. The training dataset signals with different noise factors were plotted to illustrate the impact of noise factors on the signals.

### 4.2. Model Evaluation Metrics

The mathematical formulae for these evaluation metrics can be expressed as follows:

#### Mean Squared Error (MSE)

This formula calculates the average squared difference between the observed values and the predicted values, providing a quantitative measure of the overall difference between the two signals. MSE is used to measure the difference between the original signal and the generated signal. The formula is given as follows:

$$MSE=\frac{1}{N}\sum_{i=1}^{N}{\left(x_{i}-{\hat{x}}_{i}\right)}^{2}$$

where

- $x_{i}$ is the actual value of the original signal at the ith observation.
- ${\hat{x}}_{i}\;$is the predicted or generated value of the signal at the ith observation.
- N is the total number of observations.

### 4.3. Implementation of Variational Autoencoder (VAE) Architecture

The Variational Autoencoder (VAE) is designed for unsupervised learning, particularly for generating new data points that resemble the original dataset.

**Figure 6:**
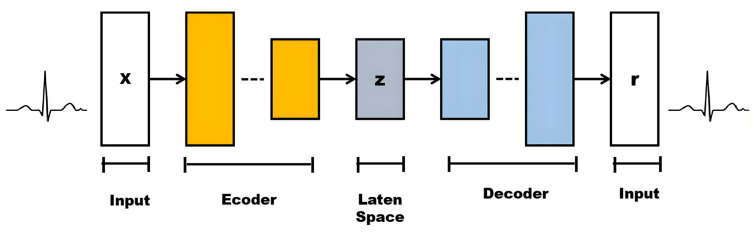
The architecture of the implemented VAE in experiments.

The VAE consists of three primary components: the encoder, the reparameterization step, and the decoder. Each component plays a crucial role in transforming input data into a latent representation and reconstructing the data from this compressed form, as shown in Figure 6.

The encoder transforms the input data x into a latent space through a series of linear transformations and activation functions as follows.

**First Linear Layer**: This reduces the input dimensionality to 128 units, followed by the ReLU activation function.

**Second Linear Layer**: This further reduces the dimensionality to 64 units, with ReLU activation.

**Third Linear Layer**: This maps the 64 units to twice the latent dimension, producing an output that is split into two parts: the mean (μ) and log variance ($log\left(\sigma {\left(x\right)}^{2}\right)$)).

The reparameterization trick enables backpropagation through the stochastic sampling process. The mean (μ) and log variance ($log\left(\sigma {\left(x\right)}^{2}\right)$) from the encoder are used to sample the latent variable z. The standard deviation is computed as σ, and z is sampled as z=μ+ε⋅σ, where ϵ is random noise from a standard normal distribution.

The decoder reconstructs the original input data from the latent variable z through three layers: the first Linear Layer maps z. to 64 units, followed by ReLU activation, the second Linear Layer transforms the output to 128 units, with ReLU activation, and the third Linear Layer maps the 128 units back to the original input dimension, producing the reconstructed output.

The forward pass process includes three steps: the first step is as follows: the input x passes through the encoder to obtain the mean (μ) and log variance ($log\left(\sigma {\left(x\right)}^{2}\right)$); the second step is as follows: the reparameterization step samples the latent variable z; and the third step is as follows: the decoder reconstructs the input from z, yielding the final output.

### 4.4. Experimental Setup and Parameter

This architecture allows the VAE to learn probabilistic mapping from the input space to a latent space and back. By training the model, the encoder learns to produce a meaningful latent representation, and the decoder learns to reconstruct the input from this representation. The VAE is effective for generating new data points similar to the training data, making it valuable in applications such as image generation, anomaly detection, and data compression. Its ability to handle high-dimensional data and learn efficient latent representations has made it a popular choice in machine learning and AI. Variational Autoencoders (VAEs) use a probabilistic framework to model data and generate new samples. The training of a VAE involves both encoder and decoder networks. Below, we explain how the inference model of a VAE can be trained through standard backpropagation and stochastic gradient descent (SGD).

Hardware and Software

○ Implement the VAE and watermarking algorithms in Python 3.12, leveraging deep learning frameworks (e.g., PyTorch 1.8, TensorFlow 2.5).
○ Use GPU acceleration for efficient model training (e.g., NVIDIA RTX GPUs).

Hyperparameters for VAE Training

○ Batch Size: Batch size is typically 16–64, depending on GPU memory.
○ Learning Rate: The learning rate is often between 1 × 10^−4^ and 1 × 10^−3^.
○ Number of Epochs: the number of epochs is 100–300, monitored by early stopping criteria.
○ Latent Dimension: This is selected based on signal complexity; common ranges are 8–64.

Watermark Parameters

○ Watermark Length: This must fit the latent representation size (for pre-reconstruction) or the number of frequency coefficients (for post-reconstruction).
○ Embedding Strength (α): This is critical for controlling the balance between watermark visibility and robustness. Typical test values are {0.1, 0.5, 0.9}.
○ Watermark Data: These could be binary patterns, textual identifiers, or random noise vectors.

Evaluation Metrics

○ Signal Fidelity:
  ▪ The MSE between original and reconstructed signals.
  ▪ PSNR or SSIM (optional) are used to evaluate signal quality more comprehensively.

○ Watermark Detection:
  ▪ The accuracy/success rate of extracting the correct watermark bits is considered.
  ▪ Threshold-based or correlation-based detection strategies are used if the watermark is noise-like.

○ Robustness Tests: Noise is applied.

Attack Scenarios

○ Introduce signal manipulations (e.g., Gaussian noise and low-pass filtering) to test whether the watermark can still be detected under realistic conditions.

### 4.5. Watermarking Experiment Results and Discussions

Table 1 shows the results of embedding watermarks on generated ECG signals using the specified model parameters. The input signal dimension (input_diminput\_diminput_dim) is 90, and the latent dimension (latent_dimlatent) is 20. The model was trained for 400 epochs with a noise factor (noise_factor) of 0. The training batch size was set to 32, and the learning rate (learning_rate) was 0.001. The watermark used was a binary series [1, 1, 1, 1, 1, 1, 1, 1, 1, 1], with a length of 10. The evaluation metric used to compare the reconstructed ECG signals to the original signals was the Mean Squared Error (MSE).

**Figure 7:**
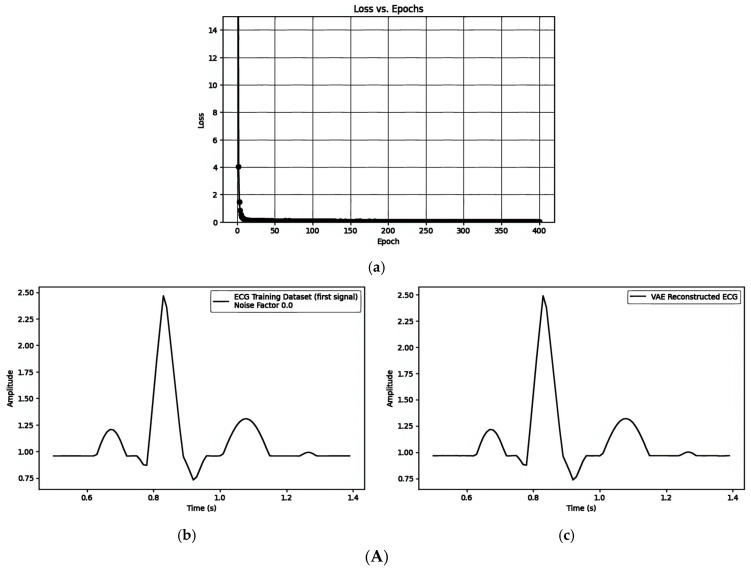

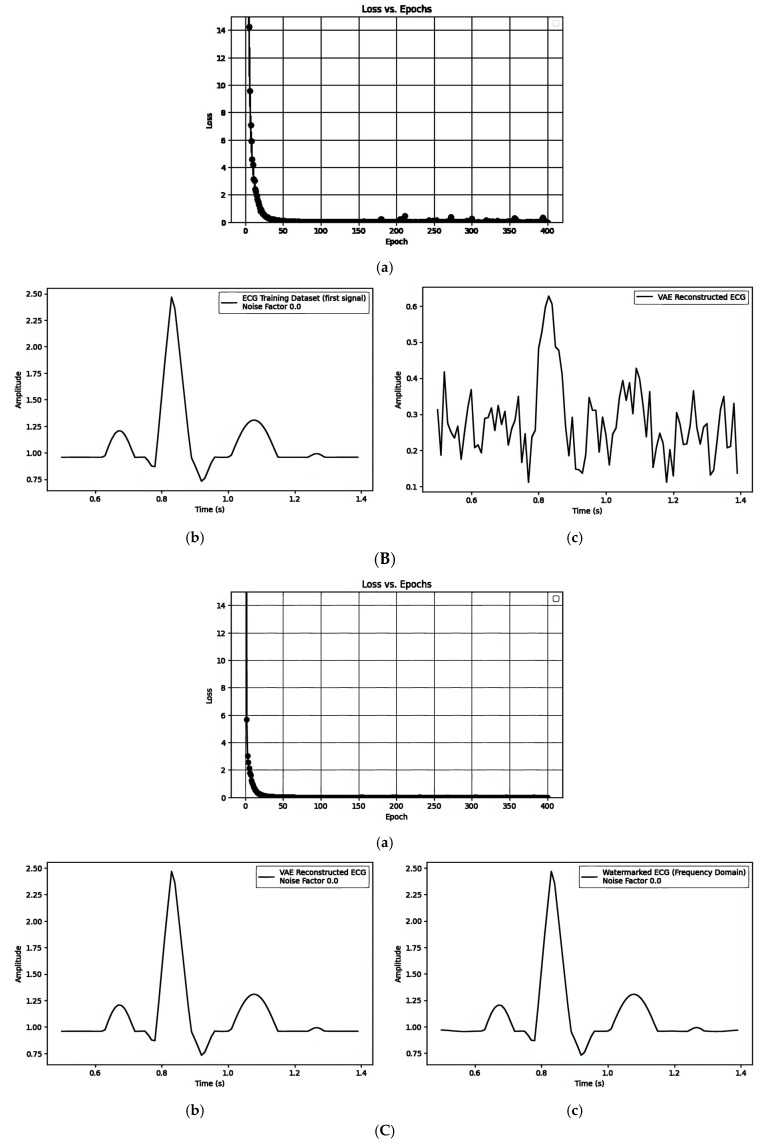
(**A**) Embedding a watermark into the mean (μ) of the VAE model: (**a**) the loss vs. epochs; (**b**) ECG training data; and (**c**) VAE model-generated ECG embedded with a watermark. (**B**) Embedding a watermark into the latent variable (z) of the VAE model: (**a**) the loss vs. epochs; (**b**) ECG training data; and (**c**) VAE model-generated ECG embedded with a watermark in the latent variable (z). (**C**) Embedding a watermark through the frequency domain: (**a**) the loss vs. epochs; (**b**) the reconstructed ECG with the VAE model; and (**c**) ECG embedded with a watermark.

**Figure 8:**
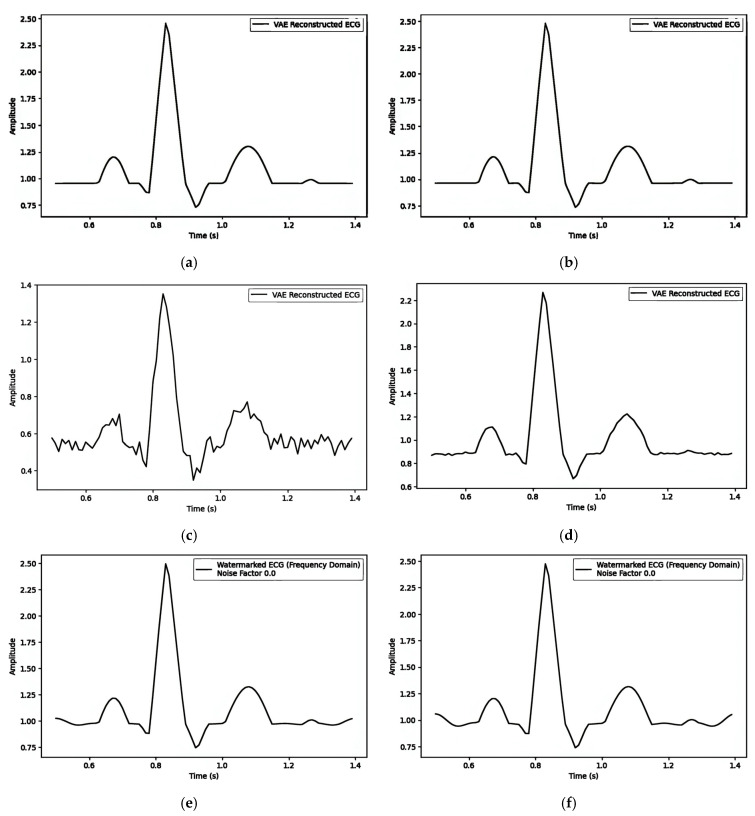
The simulated ECG at (**a**) alpha 0.5 and (**b**) alpha 0.9 embedding the watermark in the mean (μ); (**c**) alpha 0.5 and (**d**) embedding watermarks through the latent variable (z); (**e**) alpha 0.5 and (**f**) alpha 0.9 embedding watermarks through the frequency domain.

Comparing the training curve of the loss vs. epochs, Figure 7A(a),B(a),C(a) show a stable convergence curve that shows a smooth decline in loss and minimal fluctuations as it approaches the minimum loss. Figure 8a has been used for comparisons with Figure 5b with the difference between (b) and (c) and the matric MSE shown as follows in Table 2. The latent variable space embedding the watermark in the mean (μ) has the minimum distortion after watermarking.

The Mean Squared Error (MSE) values between the reconstructed ECG signals and the original training signals are influenced by the loss function used. These MSE values vary due to the reparameterization trick, which involves sampling the latent variable (z).

When comparing the evaluation metric MSE against the alpha parameter (α), the following is observed:

- Embedding the watermark in the mean (μ) results in α having an influence of less than $10^{-3}$ on the MSE.
- Embedding the watermark through the latent variable (z) results in α having an influence of less than $10^{-1}$ on the MSE.
- Embedding the watermark through the frequency domain results in α having an influence of less than $10^{-3}$ on the MSE.

This indicates that the choice of watermarking strategy and the corresponding value of α have varying impacts on the accuracy of the reconstructed ECG signals.

The latent space is a lower-dimensional space that captures the essential features of the input data. In an Autoencoder, when the encoded representation (h) has a smaller dimension than the input (x), it is referred to as an Undercomplete Autoencoder. In Table 3, all Mean Squared Error (MSE) values observed are less than $10^{-4}$, indicating that they are of the same order of magnitude. When embedding the watermark in the mean (μ), the dimension of the latent space does not have a significant influence on the MSE, preserving the visual quality of the signals. However, embedding watermarks through the latent variable (z) does affect the MSE, as the MSE values are not of the same order, indicating a potential impact on visual quality.

However, when comparing time-series data like ECG signals, relying on MSE alone can be misleading, especially if the signals are not perfectly aligned in time. MSE measures the average squared difference between corresponding points but does not account for potential time shifts. Dynamic Time Warping (DTW) distance [31] is a way to measure the similarity between two time series, even if they have different lengths or are out of sync in time. Thus, combining MSE with DTW gives a more complete picture, capturing both the differences in amplitude (MSE) and the robustness to time shifts (DTW).

#### 4.5.1. Embedding the Watermark in the Mean (μ)

**Figure 9:**
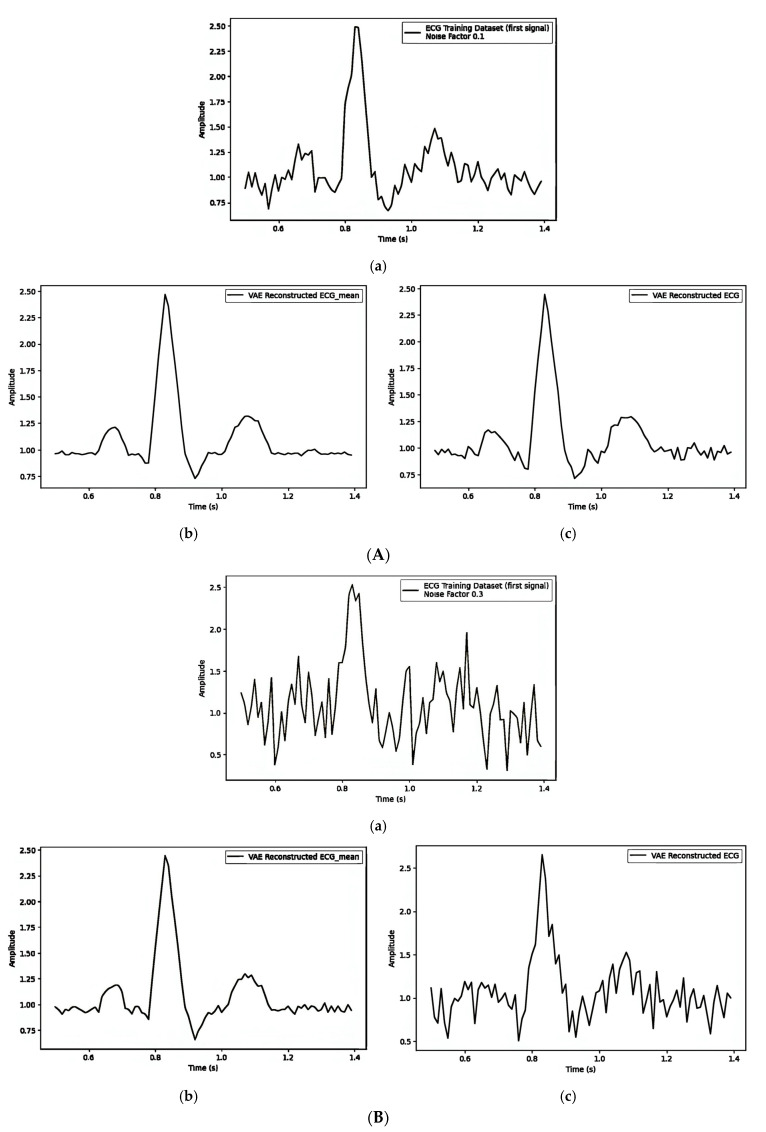

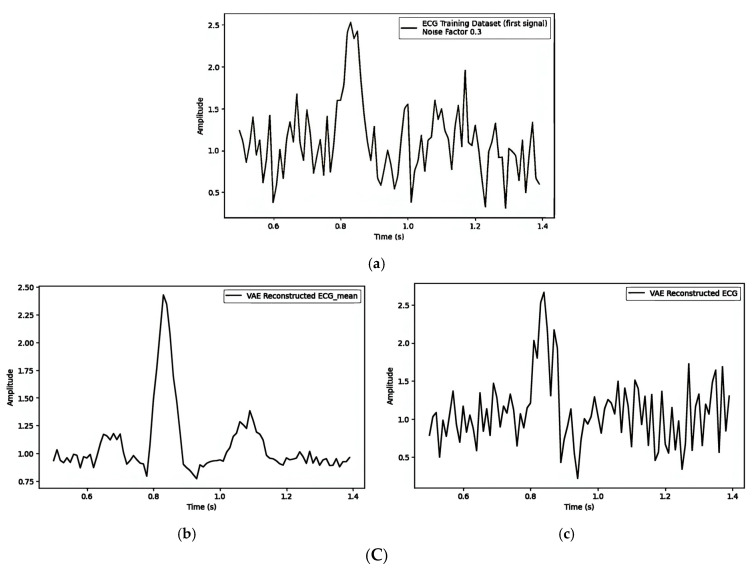
(**A**) Embedding the watermark in the mean (μ) with noise_factor = 0.1: (**a**) first original training signal; (**b**) mean of all training signals; and (**c**) VAE-reconstructed signal with watermarking. (**B**). Embedding the watermark in the mean (μ) with noise_factor = 0.3: (**a**) first original training signal; (**b**) mean of all training signals; and (**c**) VAE reconstructed signal with watermarking. (**C**) Embedding the watermark in the mean (μ) with noise_factor = 0.5: (**a**) first original training signal; (**b**) mean of all training signals; and (**c**) VAE-reconstructed signal with watermarking.

Table 4 illustrates how different noise factors impact watermark embedding in ECG signals through the mean (μ), focusing on the Mean Squared Error (MSE) and Dynamic Time Warping (DTW) distances. Figure 9A–C give separate observations for noise factors 0.1, 0.3, and 0.5, and their impact on visual quality and the quantity values are shown in Table 4. The values of MSE and DTW increase as the noise factor increases.

#### 4.5.2. Embedding the Watermark Through the Latent Variable (z)

**Figure 10:**
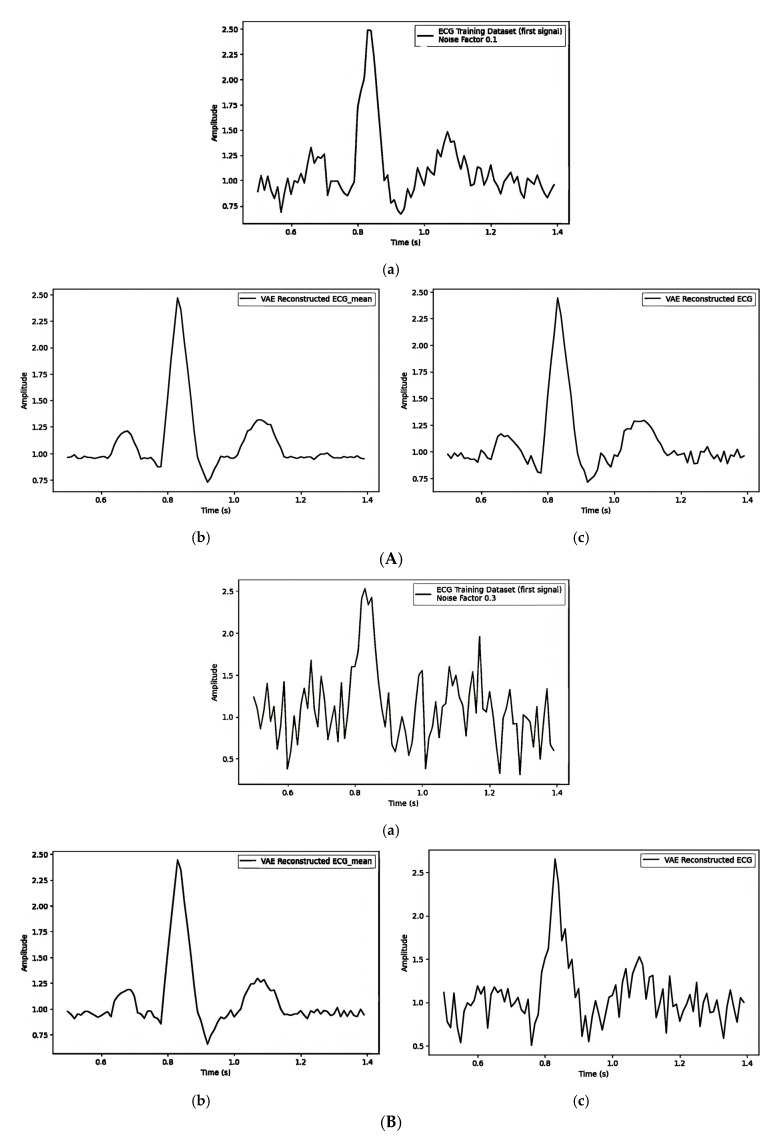

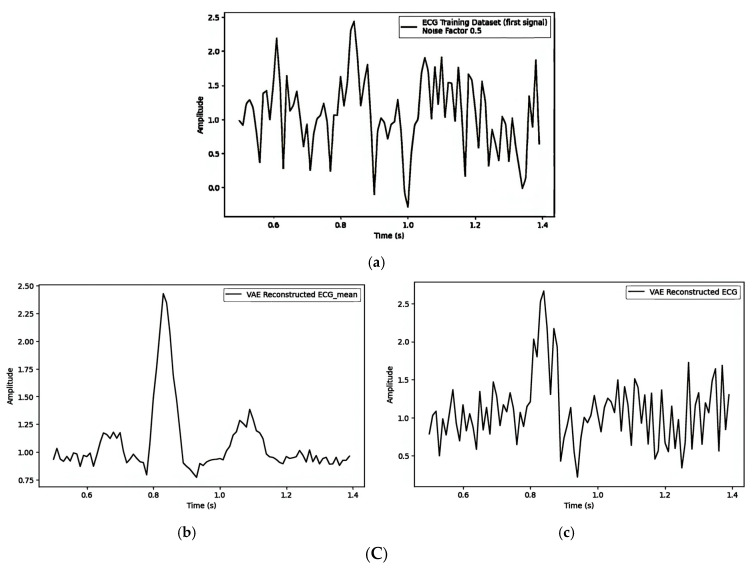
(**A**) Embedding the watermark through the latent variable (z) with noise_factor = 0.1: (**a**) first original training signal; (**b**) mean of all training signals; and (**c**) VAE-reconstructed signal with watermarking. (**B**) Embedding the watermark through the latent variable (z) with noise_factor = 0.3: (**a**) first original training signal; (**b**) mean of all training signals; and (**c**) VAE-reconstructed signal with watermarking. (**C**) Embedding the watermark through the latent variable (z) noise_factor = 0.5: (**a**) first original training signal; (**b**) mean of all training signals; and (**c**) VAE-reconstructed signal with watermarking.

Table 5 illustrates how different noise factors impact watermark embedding in ECG signals through the latent variable (z), focusing on the Mean Squared Error (MSE) and Dynamic Time Warping (DTW) distances. Figure 10A–C give separate observations for noise factors 0.1, 0.3, and 0.5, and their impact on visual quality and the quantity values are shown in Table 5. The values of MSE and DTW increase as the noise factor increases.

#### 4.5.3. Embedding Watermark Through Frequency Domain

**Figure 11:**
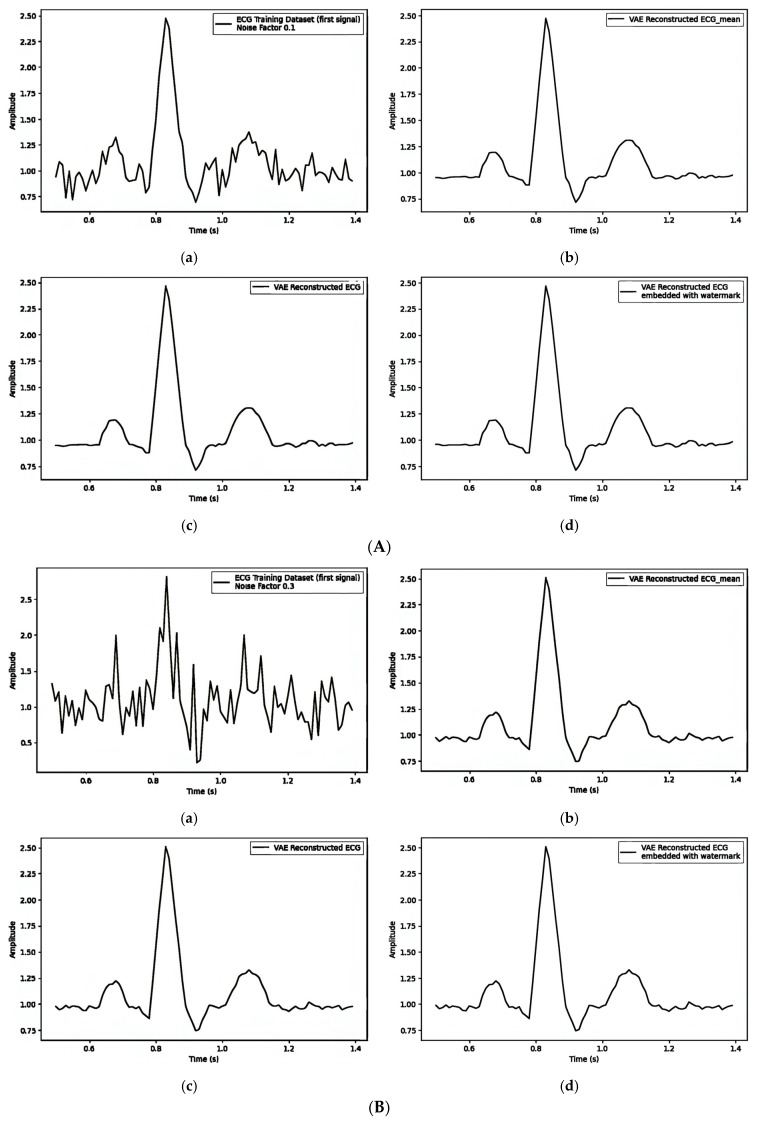

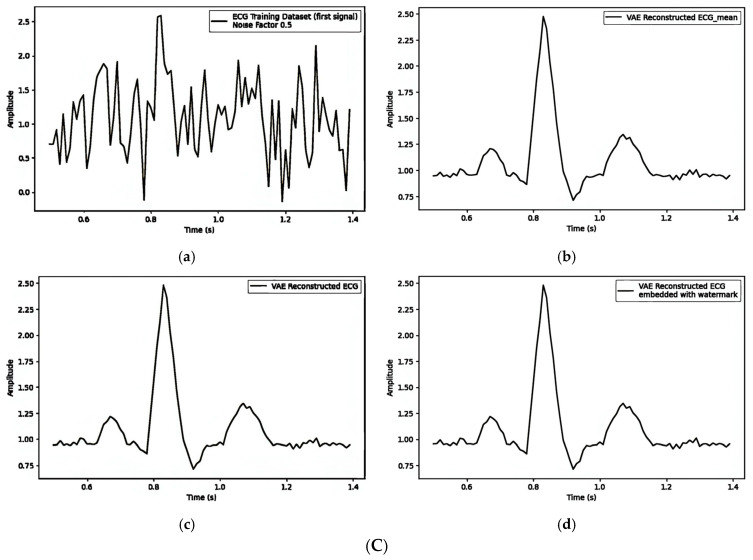
(**A**) Post-reconstruction watermarking, embedding watermarks through the frequency domain with noise_factor = 0.1: (**a**) first original training signal; (**b**) mean of all training signals; (**c**) VAE-reconstructed signal without watermarking; and (**d**) VAE-reconstructed signal with watermarking. (**B**) Post-reconstruction watermarking, embedding watermarks through the frequency domain with noise_factor = 0.3: (**a**) first original training signal; (**b**) mean of all training signals; (**c**) VAE-reconstructed signal without watermarking; and (**d**) VAE-reconstructed signal with watermarking. (**C**) Post-reconstruction watermarking, embedding watermarks through the frequency domain with noise_factor = 0.5: (**a**) first original training signal; (**b**) mean of all training signals; (**c**) VAE reconstructed signal without watermarking; and (**d**) VAE reconstructed signal with watermarking.

Table 6 illustrates how different noise factors impact watermark embedding in ECG signals through the frequency domain, focusing on the Mean Squared Error (MSE) and Dynamic Time Warping (DTW) distances. Figure 11A–C give separate observations for noise factors 0.1, 0.3, and 0.5, and their impact on visual quality and the quantity values are shown in Table 6. The values of MSE and DTW increase as the noise factor increases.

In general, transparency is the key performance of steganography. It is usually measured by the signal-to-noise ratio (SNR). *n* represents the number of testing samples of an ECG signal; $s_{i}\;$ represents the original ECG signal; and ${\hat{s}}_{i}$ represents the embedded (or hidden) ECG signal. SNR is defined as follows:

(7) $$SNR=-10\log_{10}\left[\frac{\sum_{i=1}^{n}{\left({\hat{s}}_{i}-s_{i}\right)}^{2}}{\sum_{i=1}^{n}s_{i}^{2}}\right]$$

The extraction rate of the hidden message is another key performance of steganography. It is usually measured by the bit-error-rate (BER), which is defined by the following:

BER = (error bits/total bits) × 100% (8)

In Table 7, we compare the performance of the proposed method with references [32,33]. From the results of Table 7, one can observe that the proposed method has a good SNR and low BER.

For the evaluation of the watermarking algorithm, synthetic noise (Gaussian noise) is added. The experiments introduce Gaussian noise at various signal-to-noise ratio (SNR) levels to simulate artifacts and interference in ECG signals. Specifically, zero-mean Gaussian noise with a controlled variance ($\sigma^{2}$) is added to the watermarked ECG waveforms at predefined SNRs. This approach mirrors common noise sources in clinical settings, such as electrical interference and motion artifacts, allowing for an objective assessment of each watermarking method’s robustness. By systematically varying σ\sigma, the experiments capture a range of mild-to-moderate distortions, providing insight into how well the proposed techniques maintain signal fidelity and preserve watermark detectability under increasing noise levels.

The analysis reveals that watermark embedding in ECG signals through the mean (μ), latent variable (z), and the frequency domain maintains low Mean Squared Error (MSE) values and signal fidelity across various watermarking strengths (α), latent space dimensions, and noise factors. Embedding watermarks through the latent variable (z) exhibits increased sensitivity to watermarking strength, latent space dimensions, and noise factors. Higher distortion is observed with increased α values, reduced latent space dimensions, and greater noise levels, indicating a trade-off between watermark strength and signal quality. This study highlights that watermark embedding through the mean (μ) and frequency domain offers more stable performance, while the latent variable (z) approach requires careful parameter tuning to manage distortion effectively. Understanding the trade-offs between these strategies assists in selecting the most effective approach for digital content protection. Ultimately, integrating VAEs with watermarking provides a robust solution for safeguarding and verifying digital content, balancing watermark invisibility and signal quality.

The experimental results show that embedding watermarks into the mean (μ) and using frequency-domain methods consistently maintain low MSE values and preserve ECG signal fidelity under varying watermarking strengths (α), latent space dimensions, and noise levels. In contrast, embedding through the latent variable (z) is more sensitive to these factors, often exhibiting higher distortion at larger α, smaller latent dimensions, or under increased noise. While μ- and frequency-based approaches require less parameter tuning and yield more stable performance, the z-based method offers finer control but demands careful calibration to avoid degrading the ECG waveform. Overall, these findings highlight the trade-offs among invisibility, robustness, and computational complexity, underscoring the importance of tailoring watermarking strategies to specific applications for effectively safeguarding and verifying digital ECG content.

In this paper, we systematically compared the performance of three watermarking strategies: embedding watermarks in the mean (μ) of the VAE’s latent space, through the latent variable (z), and using post-reconstruction watermarking in the frequency domain. Our results demonstrate that embedding watermarks through the mean (μ) and in the frequency domain achieves a low Mean Squared Error (MSE) while maintaining stable signal fidelity across varying watermark strengths (α), latent space dimensions, and noise levels. These methods offer the merits of robust performance and minimal impact on signal quality. In contrast, embedding watermarks through the latent variable (z) exhibits increased sensitivity to these factors, requiring careful parameter tuning to avoid signal degradation. Overall, our analysis highlights the trade-offs between invisibility, robustness, and computational complexity, providing insights into the selection of the most appropriate watermarking technique for ECG sensor applications.

**The important architectural constraint is the fixed latent dimension.** The chosen latent dimension (e.g., 16) may not universally capture all clinically relevant nuances of different ECG morphologies. An insufficient latent dimension could lead to information bottlenecks that degrade both reconstruction fidelity and watermark robustness. Conversely, excessively large latent spaces risk overfitting and may inflate computational costs, limiting practical deployment.

**The hyperparameter limitations include** β **-VAE balancing, learning rate, and batch size.**

β**-VAE Balancing**: The trade-off parameter β (balancing KL Divergence and reconstruction loss) significantly influences how the latent representation is learned. Small β values favor faithful reconstructions at the risk of weaker regularization; large β values enhance disentanglement but may reduce reconstruction quality. Either extreme could hinder the effectiveness or invisibility of embedded watermarks.

**Learning Rate and Batch Size**: Common hyperparameters like the learning rate ($10^{-4}$–$10^{-3}$) and batch size (16–64) heavily influence training stability. Suboptimal choices may lead to the disappearance or explosion of gradients, producing inconsistent watermark results or preventing the model from converging to a robust solution.

## 5. Conclusions

Designing an ECG sensor circuit necessitates detecting, amplifying, filtering, and conditioning weak heart signals, and its performance can be assessed by generating diverse, watermarked ECG data. This study advances digital watermarking by applying VAE-generated signals to validate sensor functionality, emphasizing the need for robust data security in modern healthcare. Experimental comparisons of embedding methods—mean (μ), latent variable (z), and frequency domain—demonstrate that the mean (μ) and frequency domain approaches consistently deliver low distortion and reliable watermark protection, whereas the z-based technique requires careful tuning to avert signal degradation. Future research should explore optimizing latent variable embedding to boost resilience, conduct thorough noise-sensitivity analyses for post-reconstruction watermarking, and investigate hybrid systems that merge the mean (μ) and frequency domain methods for an optimal balance of invisibility and signal fidelity.

The waveforms utilized in this manuscript have inherent limitations due to these noise sources. Recognizing these constraints is essential, as they can affect the accuracy of ECG analysis. Therefore, further research is warranted to explore the impact of different noise sources on ECG analysis and to develop advanced noise reduction techniques that preserve the integrity of the original cardiac signals. Future research could investigate more sophisticated generative models—such as hybrid VAEs, GAN-VAEs, or Transformers—to improve watermark invisibility and robustness. These architectures may better capture the fine-grained nuances of ECG signals, leading to higher-fidelity reconstructions and more secure watermark embeddings.

## Figures and Tables

**Table 1:** The parameters of the model and data for the figures.

Figure 7A(a–c)/Figure 7B(a–c)/Figure 7C(a–c)						
Model Parameters					Data Parameters	
Input dimension	Latent dimension	Learning rate	Batch size	Training epochs	Noise factor	Watermark length
90	20	0.001	32	400	0.0	10

**Table 2:** MSE (visual quality) vs. alpha (watermarking strength) for three watermarking strategies embedding watermarks [1, 1, 1, 1, 1, 1, 1, 1, 1, 1.] with noise factor 0.0.

Watermarking Strategies	MSE	Comprison Figures	alpha
1. **Pre-Reconstruction Watermarking:** Embedding the watermark in the mean (μ)	0.00025240	Figure 7A(b) and Figure 7A(c)	0.1
	0.00002263	Figure 5b and Figure 8a	0.5
	0.00002474	Figure 5b and Figure 8b	0.9
2. **Pre-Reconstruction Watermarking:** Embedding watermarks through the latent variable (z)	0.71435501	Figure 7B(b) and Figure 7B(c)	0.1
	0.25729716	Figure 5b and Figure 8c	0.5
	0.00806380	Figure 5b and Figure 8d	0.9
3. **Post-Reconstruction Watermarking:** Embedding watermarks through the frequency domain	0.00000679	Figure 7C(b) and Figure 7C(c)	0.1
	0.00016975	Figure 5b and Figure 8e	0.5
	0.00055000	Figure 5b and Figure 8f	0.9

**Table 3:** MSE vs. dimension of latent space for two watermarking strategies embedding watermarks [1, 1, 1, 1, 1, 1, 1, 1, 1, 1.] with noise factor 0.0 and alpha 0.1.

Watermarking Strategies	MSE	Dimension of Latent Space
1. **Pre-Reconstruction Watermarking:** Embedding the watermark in the mean (μ)	0.00002229	30
	0.00002773	20
	0.00003235	10
2. **Pre-Reconstruction Watermarking**: Embedding watermarks through the latent variable (z)	0.00000756	30
	0.00030506	20
	0.01020047	10

**Table 4:** Noise factor influence of embedding the watermark in the Mean (μ) in latent space.

Noise Factor	Figure 9A/Figure 9B/Figure 9C					
	MSE			DTW		
	(a) vs. (b)	(a) vs. (c)	(b) vs. (c)	(a) vs. (b)	(a) vs. (c)	(b) vs. (c)
0.1	0.0098	0.0122	0.0016	8.85	9.12	3.67
0.3	0.1033	0.1736	0.0494	23.37	24.04	17.42
0.5	0.2260	0.3738	0.1063	38.48	38.60	25.79

**Table 5:** The influence of noise factor on embedding the watermark through the latent variable (z).

Noise Factor	Figure 10A/Figure 10B/Figure 10C					
	MSE			DTW		
	(a) vs. (b)	(a) vs. (c)	(b) vs. (c)	(a) vs. (b)	(a) vs. (c)	(b) vs. (c)
0.1	0.0823	0.0969	0.0216	22.63	20.92	12.32
0.3	0.0918	0.1018	0.0102	23.76	21.92	9.06
0.5	0.2639	0.4514	0.1599	37.45	34.80	28.18

**Table 6:** Noise factor influence of post-reconstruction watermarking embedding watermark through frequency domain.

Noise Factor	Figure 11A/Figure 11B/Figure 11C					
	MSE			DTW		
	(a) vs. (b)	(a) vs. (c)	(b) vs. (c) (b) vs. (d) (c) vs. (d)	(a) vs. (b)	(a) vs. (c)	(b) vs. (c) (b) vs. (d) (c) vs. (d)
0.1	0.00769329	0.00768736	0.00000606	7.36723554	7.38519794	0.35533977
			0.00000849			0.31853223
			0.00000679			0.17227793
0.3	0.09544947	0.09547500	0.00000714	22.89780141	22.87669791	0.38388956
			0.00001911			0.52939296
			0.00000679			0.20855498
0.5	0.22180541	0.22261526	0.00001820	37.97530274	37.92291443	0.58136564
			0.00002480			0.67344922
			0.00000679			0.22654438

**Table 7:** Performance comparison between the proposed method and other methods.

Data ID	Reference [32] (Time Domain)		Reference [33] (DWT Domain)		Proposed Method (Time Domain)	
	SNR	BER (%)	SNR	BER (%)	SNR	BER (%)
1	51.7	23.2	38.4	25.1	50.4	0
2	68.3	21.1	32.7	24.3	52.3	0
3	60.9	23.7	34.2	23.8	50.6	0
4	55.7	24.5	37.4	24.6	51.3	0
5	38.4	25.3	32.5	25.3	48.5	0
6	29.2	20.7	26.9	23.7	42.6	0
7	63.6	22.8	26.7	26.2	53.2	0
8	40.3	24.1	41.9	24.5	48.5	0

## Data Availability

Data are contained within the article.
